# Co-Infection of Pulmonary Aspergillosis and Cryptococcal Meningitis in an HIV-Positive Patient: A Case Report

**DOI:** 10.1155/crdi/5630156

**Published:** 2025-08-05

**Authors:** Marjan Hemmatian, Sadegh Khodavaisy, Hossein Kazemizadeh, Behnaz Jahanbin, Reza Ershadi, Maryam Moradi, Mohammadreza Salehi, Jianping Xu, Megan Hitchcock, Kazem Ahmadikia, Ali Ahmadi, Seyed Ali Dehghan Manshadi

**Affiliations:** ^1^Department of Infectious Diseases and Tropical Medicine, Iranian Research Center HIV/AIDS (IRCHA), Tehran University of Medical Science, Tehran, Iran; ^2^Department of Medical Parasitology and Mycology, Tehran University of Medical Sciences, Tehran, Iran; ^3^Department of Biology, McMaster University, Hamilton, Ontario L8S 4K1, Canada; ^4^Advanced Thoracic Research Center, Department of Internal Medicine, Imam Khomeini Hospital Complex, Tehran University of Medical Sciences, Tehran, Iran; ^5^Cancer Institute, Pathology Department, Imam Khomeini Hospital Complex, Tehran University of Medical Sciences, Tehran, Iran; ^6^Department of Thoracic Surgery, Mahdi Hospital, Tehran University of Medical Sciences, Tehran, Iran; ^7^Eye Research Center, The Five Senses Health Institute, Rasoul Akram Hospital, Iran University of Medical Sciences, Tehran, Iran; ^8^Department of Medical Parasitology and Mycology, School of Medicine, Shahid Beheshti University of Medical Sciences, Tehran, Iran

**Keywords:** cryptococcal meningitis, HIV, opportunistic fungal infection, pulmonary aspergillosis

## Abstract

Opportunistic fungal infections (OFIs) are common among human immunodeficiency virus (HIV) –positive patients, especially in those with delayed diagnosis and treatment. Patients with severe HIV/AIDS with clusters of differentiation 4 (CD4) counts less than 100 are significantly prone to develop multiple OFIs. In the current study, we present a case of co-infection of pulmonary aspergillosis and cryptococcal meningitis in a late-diagnosed HIV patient with a low CD4 count.

## 1. Introduction

Human immunodeficiency virus (HIV) substantially predisposes patients to a variety of opportunistic infections, including fungal infections. Pneumocystosis, candidiasis, cryptococcosis, and histoplasmosis are the most frequent fungal infections observed among HIV-positive patients. Although the incidence of opportunistic infections is declining in response to the broad adoption of highly active antiretroviral therapy (HAART), fungal infections are still a great concern among immunocompromised patients [1, 2]. Cryptococcosis is an invasive fungal infection estimated to be the second-leading cause of death among HIV/AIDS patients [3]. This infection is caused by yeasts in the pathogenic *Cryptococcus* species complex, which was recently classified as a critical priority fungal pathogen by the World Health Organization (WHO) [4, 5]. Though often fatal, aspergillosis, an infection most frequently caused by the mold *Aspergillus fumigatus*, has not been considered a major opportunistic infection among HIV/AIDS patients since 1984. Nonetheless, aspergillosis and other invasive mold infections are observed among HIV-positive patients who undergo neutropenia or chronic corticosteroid therapy [6, 7]. Although opportunistic fungal infections (OFIs) are common among HIV-positive patients, co-fungal infections are rarely reported. In this report, we describe the clinical and paraclinical characteristics of an HIV-positive patient without a history of neutropenia, who developed both pulmonary aspergillosis (PA) and cryptococcal meningitis within 1 month.

## 2. Case Presentation

**Figure 1:**
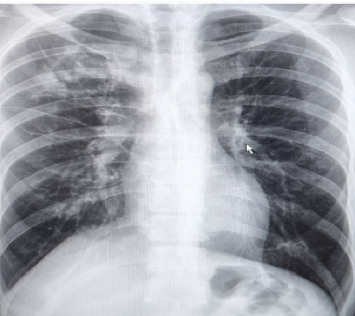
The patient's chest X-ray.

**Figure 2:**
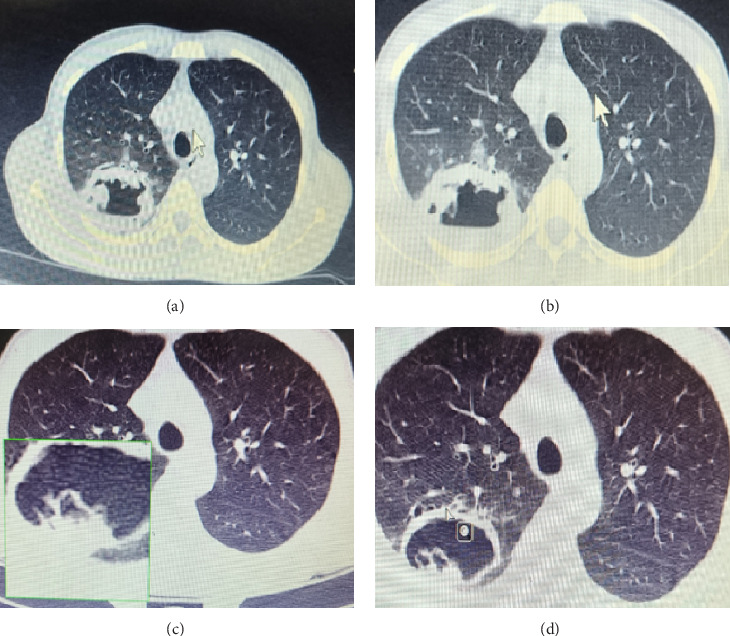
The patient's chest CT scan. (a) and (b) A thick-walled 48 ^∗^ 34 mm cavity with an air-fluid level in the posterior segment of the right upper lobe (RUL). (c) and (d) Suggested hydatid cyst.

A 46-year-old homosexual man was presented to the emergency ward with a productive cough, fever, weight loss, and oral candidiasis reported in the three months before his visit. Considering the risk factors, an infectious disease specialist was consulted, and a rapid lateral flow assay (LFA) HIV test was requested and returned positive. HIV infection was confirmed with a fourth-generation ELISA. Initial immunologic staging showed a cluster of differentiation 4 (CD4) count of 41 cells/μL. The patient was conscious and oriented for baseline physical examination with the following vital signs: a fever with 38 °C body temperature, a heart rate of 100/min, a respiratory rate of 20/min, a blood pressure of 90/60 mmHg, and an oxygen saturation level of 94%. The chest computed tomography (CT) findings were clear, and no lymphadenopathy, hepatosplenomegaly, or skin lesions were found. The baseline laboratory test results of the patient's blood are summarized in Table 1. On the first day of admission, intravenous piperacillin-tazobactam 4.5 mg every 6 h alongside a 4-drug regimen of antituberculosis (TB) was initiated (isoniazid 300 mg/daily, rifampin 600 mg/daily, pyrazinamide 1500 mg/daily, and ethambutol 800 mg/daily). A chest X-ray and CT scan of the chest revealed a cavitary lesion in the posterior segment of the right upper lobe (Figures 1 and 2(a), 2 (b), 2 (c), and 2(d)). Therefore, a 3x sputum smear for TB diagnosis, TB GeneXpert, and serum cryptococcal antigen (CrAg) test were requested, all of which tested negative. Nonetheless, because of the cavitary lesion in the lung, bronchoscopy and bronchoalveolar lavage (BAL) were performed alongside TB-specific PCR to further rule out TB and other microbial etiologies. The tests were negative for both bacterial and fungal agents in the BAL specimen (Table 2). Despite anti-TB and antibiotic therapy, the patient's clinical condition did not show improvement. Therefore, the chest CT scan was repeated. Based on the second CT scan and radiologic consultation, a hydatid cyst was suspected. The serum *Echinococcus* antigen and antibody were both reported as negative. Nineteen days after admission, intravenous piperacillin-tazobactam and anti-TB regimen were discontinued, and Truvada (a combination of emtricitabine and tenofovir disoproxil fumarate), dolutegravir, and albendazole were started.

**Figure 3:**
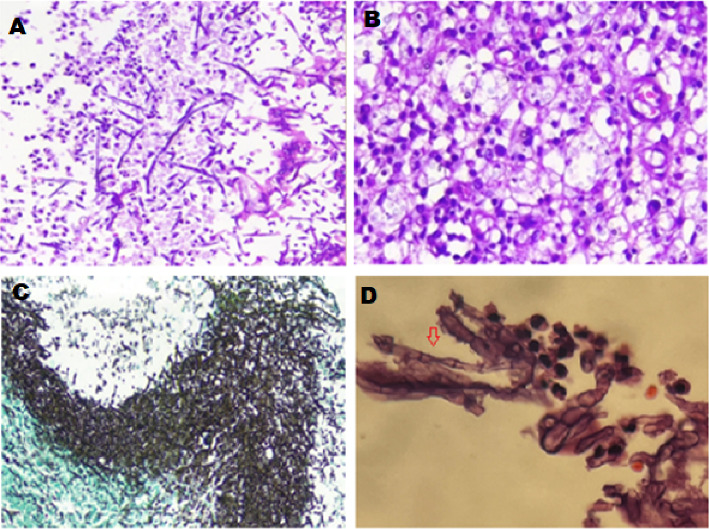
Pathological examination of the lung segmentomy specimen showing both septate fungal hyphae and yeast cells. (A) and (B) Hematoxylin and eosin (H&E) stain. (C) and (D) Grocott–Gomori's methenamine silver (GMS) stain. Lung parenchyma with necrosis and invasive fungal elements consisting of nonpigmented narrow branching septate hyphae and a collection of yeasts and hyphae in alveolar macrophages. Collection of foam macrophages in alveolar spaces and a mild PMN-dominant inflammatory reaction around the fungus ball.

**Figure 4:**
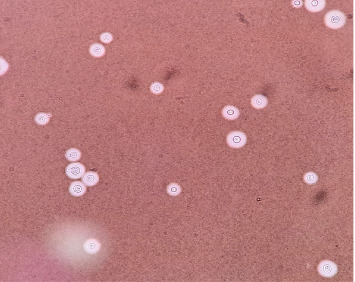
A distinct capsule surrounding *C. neoformans* yeast cells is visible in the CSF smear stained with India ink.

In the following days, a gradual deterioration with the onset of new respiratory signs was observed. Based on this decline, the decision for the patient to undergo a thoracotomy and segmentectomy was made. Surgery found the macroscopic appearance of the lesion not consistent with a hydatid cyst. Pathological examination with hematoxylin and eosin (H&E) of the lung biopsy revealed nonpigmented narrow branching septate hyphae and yeast with budding cells in alveolar macrophages. Also, Grocott–Gomori's methenamine silver (GMS) stain was used to indicate septate hyphae character and whether the yeast forms could represent *Cryptococcus* or another yeast pathogen in the lung biopsy. Stepwise differential diagnoses of aspergillosis, fusariosis, and *Pseudallescheria* species were performed (Figures 3(A), 3(B), 3(C), and 3(D)). The first step in fungal differential diagnosis used *Aspergillus*-specific PCR, which presented positive. Therefore, albendazole was discontinued, and treatment shifted to oral voriconazole 200 mg every 12 h. After 1 month of hospitalization, the patient was discharged and continued with HAART therapy and voriconazole treatment. After 2 weeks, the patient reported severe headache, restlessness, shivering, fever, and blurred and double vision. Neurological and ophthalmological exams were all normal. The patient underwent a brain CT scan with and without contrast to rule out brain abscesses or mass lesions, followed by a lumbar puncture (LP) (Table 3 and Figure 4). Encapsulated yeast cells were detected on direct examination stained with Indian ink (Figure 4). CrAg detection using LFA and direct molecular detection by the 21-plex PCR method on CSF samples was positive for cryptococcosis, which all yielded consistent proof for cryptococcal meningitis. The isolated *Cryptococcus* strain (TMML 3988) underwent multilocus sequence typing, which is a common method used to identify *Cryptococcus* by analyzing portions of seven housekeeping genes (*CAP59*, *GPD1*, *LAC1*, *PLB1*, *SOD1*, *URA5*, and *IGS1*) [8]. Examination of the URA5 gene identified the isolate as *Cryptococcus neoformans* [9]. Due to antigen properties, this species of *Cryptococcus* is commonly referred to as Serotype A and is the most clinically relevant species of *Cryptococcus* worldwide [10]. This isolate was further designated as *C. neoformans* molecular type VNI. This specific group of *Cryptococcus* is reportedly responsible for ∼95% of all cryptococcal infections worldwide [11]. The allele type (AT) at each of the seven MLST loci was determined (*CAP59* = *AT7*, *GPD1* = *AT5*, *LAC1* = *AT3*, *PLB1* = *AT3*, *SOD1* = *AT1*, *URA5* = *AT1*, and *IGS1* = *AT1*). Based on these results, the complete MLST profile was matched to sequence type (ST) ST69 [12]. Accordingly, 5 mg/kg of liposomal amphotericin B plus 800 mg of fluconazole was started for the patient due to the lack of 5-fluorocytosine access in Iran. The patient's clinical condition deteriorated, and 24 h later, the patient unfortunately died.

## 3. Discussion

HIV-positive patients, specifically those with a CD4 count less than 200, are at manifold risk of developing fungal infections. In this population, fungal infections are the major cause of morbidity and mortality. The clinical form of OFI in HIV-positive patients ranges from superficial involvement such as oral candidiasis to disseminated forms such as cryptococcosis [13]. Among fungal infections, aspergillosis is a rare complication in HIV-positive patients and is primarily associated with HIV-positive individuals experiencing co-factors, including neutropenia, chronic corticosteroid use, pneumocystis, CMV infection, CD4 count < 100 cells/mm3, and delayed treatment. The most common presentation of HIV-associated aspergillosis occurs in the lungs. However, involvement has also been reported with the central nervous system (CNS), heart, kidneys, and sinuses [14]. Our patient who presented with a cavitary lesion, productive cough, and fever responded well to voriconazole. Therefore, in HIV-positive patients, specifically those with a profoundly diminished CD4 count, cough, fever, and cavitary lung lesion, aspergillosis should be considered in the differential diagnosis [7]. To our knowledge, this is the first report of ST69 within Iran and the second report within the Middle East, with a previous report of ST69 identified in Kuwait. In addition, ST69 is the ninth most commonly reported ST worldwide, identified in parts of Europe, Asia, Africa, and South America [15]. The incidence of aspergillosis in a large-scale study of HIV-infected patients was observed to be 3.5 cases per 1000 person-years. AIDS-associated aspergillosis is typically accompanied by a low CD4 count (less than 100 cells/mm^3^), and in about half of the HIV-associated aspergillosis cases, there is concurrent neutropenia or use of corticosteroid therapy. In the remaining cases, advanced AIDS is the only apparent risk factor. Since the isolation of *Aspergillus* from respiratory secretions does not reliably indicate invasive PA in AIDS patients, a histopathological diagnosis is usually necessary to verify the condition. Unfortunately, the prognosis for HIV-infected patients with PA is generally poor, with a median survival time of 3 months following diagnosis. As observed in this case, an increase in size or number of lesions detected by chest radiographs/CT, elevated serological biomarkers (antibody or antigen), and large volume hemoptysis are often associated with poor prognosis of PA [14]. CM is a critical neurological infection typically associated with advanced HIV/AIDS and is responsible for 15% of mortality among HIV/AIDS patients [16, 17]. Based on the time of HAART initiation and the disease presentation, we believed that unmasking immune reconstitution inflammatory syndrome (IRIS) was responsible for our patient's cryptococcal meningitis. Despite the low probability, it is necessary to consider co-infection in HIV-positive patients who do not show any improvement with standard treatments or patients who present new symptoms or exacerbation [18]. The local stigma that exists toward homosexual patients and the lack of awareness lead to late diagnosis and treatment of HIV and put the patients at manifold risk for serious complications such as life-threatening opportunistic infections. Therefore, it is essential to identify and educate these susceptible groups of patients. The WHO and IDSA guidelines highly urge CrAg screening for HIV-positive individuals with CD4 levels of ≤ 100 cells/μL [19, 20]. Preemptive CrAg screening remains a critical strategy to reduce mortality, but in some settings, such as Iran, implementation is limited due to resource constraints and the lack of routine screening in national HIV programs.

In conclusion, the detection of several systemic mycoses demonstrates the vulnerability of advanced HIV-positive patients. The introduction of HAART and primary prophylaxis for opportunistic infections has improved the quality of life in these individuals.

## Figures and Tables

**Table 1:** Laboratory test results of the patient on admission.

Blood parameter	Value/concentration	Blood parameter	Value/concentration	Blood parameter	Result/concentration
WBC	7700 (92% of PMN and 5% of lymphocytes) (4500–11,000 WBCs per µL)^∗^	Na	130 (135–145 mEq/L)	ALT	Normal
HB	9 (14–18 g/dL)	K	4 (3.6–5.2 mmol/L)	AST	Normal
PLT	166,000 (150,000–450,000 platelets per microliter of blood)	Mg	2 (1.7–2.2 mg/dL)	ALP	Normal
BUN	**37** (5–20 mg/dL)	P	**2** (2.8–4.5 mg/dL)	LDH	**560** (140–280 U/L)
Cr	1 (0.7–1.3 mg/dL)	Ca	**8.3** (8.6–10.3 mg/dL)	Direct bilirubin	Normal
FBS	**111** (70–100 mg/dL)	Albumin	3.5 (3.4–5.4 g/dL)	Total bilirubin	Normal
PT	12.7 (10–13 s)	Uric acid	4.2 (3.5 and 7.2 mg/dL)	GGT	Negative
PTT	25 (25–35 s)	ESR	**110** (less than 15 mm/h)	Amylase and lipase	Normal
INR	1.04 (2.0–3.0)	CRP	**114** (0.3–1.0 mg/dL)	IGRA	Negative

*Note:* ALP = alkaline phosphatase; ALT = alanine aminotransferase; AST = aspartate aminotransferase; Ca = calcium; Cr = creatinine; HB = hemoglobin; K = potassium; LDH = lactate dehydrogenase; Mg = magnesium; Na = sodium; P = phosphor; PLT = platelet count. The bold values indicate laboratory results that are outside the normal reference range.

Abbreviations: BUN = blood urea nitrogen; CRP = C-reactive protein; ESR = erythrocyte sedimentation rate; FBS = fasting blood sugar; GGT = gamma-glutamyl transpeptidase; IGRA = interferon gamma release assay; INR = international normalized ratio; PT = prothrombin time; PTT = partial thromboplastin time; WBC = white blood cell.

^∗^Normal ranges are provided within the parentheses.

**Table 2:** Results of the microbial, serological, and molecular assays performed on the bronchoalveolar lavage specimen.

Test parameter	Result	Test	Result
RBC	0–1	*Nocardia* PCR	Negative
WBC	0–1	Fungal elements	Negative
Bacteria	Not seen	Fungal culture	Negative
Bacterial growth	Negative	Panfungal PCR	Negative
Cytology	Negative	*Mycobacterium* smear	Negative
Galactomannan	0.3	*Mycobacterium* PCR	Negative

**Table 3:** Results of CSF laboratory analysis.

Test parameter	Result
Appearance	Semiclear and colorless
CSF pressure	High
Glucose	26 (the BS was 102 at the time of examination)
RBC	**260** (0–5)^∗^
WBC	**8** (0–5)
Adenosine deaminase (ADA)	3
Protein	**120** (< 45 mg/dL)
MTB smear and PCR	Negative
CMV PCR	Negative
India ink preparation	Encapsulated yeast
Fungal culture	*Cryptococcus* spp.
Cryptococcal polysaccharide antigen	Positive (+++)
Cryptococcal PCR	*Cryptococcus neoformans*
Bacterial culture	Negative

*Note:* The bold values indicate laboratory results that are outside the normal reference range.

^∗^Normal ranges are provided within parentheses.

## Data Availability

All data generated or analyzed during this study are included in this published article. However, more data are available from the authors upon reasonable request.
